# T-cell exhaustion in tumor immunology: mechanisms, heterogeneity, and therapeutic strategies

**DOI:** 10.3389/fimmu.2026.1841281

**Published:** 2026-05-14

**Authors:** Ruoxuan Wang, Yingjie Guo

**Affiliations:** 1 The Fourth Clinical Medical College of Harbin Medical University, Harbin Medical University, Harbin, China; 2 Arc Institute, Stanford University, Palo Alto, CA, United States

**Keywords:** immunology, mechanism analysis, T-cell, therapeutic strategies, tumor

## Abstract

T-cell exhaustion is a central mechanism limiting the durability of antitumor immunity and the long-term efficacy of cancer immunotherapy. Arising under persistent antigenic stimulation and sustained microenvironmental stress, exhausted CD8^+^ T cells undergo progressive functional impairment accompanied by stable transcriptional, epigenetic, and metabolic reprogramming. Importantly, exhaustion is now understood not as a uniform dysfunctional endpoint but as a hierarchically organized and context-dependent differentiation continuum comprising progenitor, intermediate, and terminally exhausted states with distinct degrees of plasticity and therapeutic responsiveness. This framework helps explain why immune checkpoint blockade and related therapies often produce incomplete and non-durable clinical responses, as they predominantly act on progenitor-like exhausted T cells while leaving terminally exhausted populations largely refractory to reprogramming. In this review, we integrate current knowledge of the developmental heterogeneity, molecular mechanisms, and tumor microenvironmental regulation underlying T-cell exhaustion, and examine how these features shape the efficacy of major immunotherapeutic strategies. We further suggest that future progress will depend on moving beyond attempts to globally reverse exhaustion and instead adopting state-oriented approaches that preserve progenitor-like T-cell pools, restrain terminal differentiation, and remodel the immunosuppressive tumor microenvironment.

## 1 Introduction

T lymphocytes are the principal effector cells responsible for immune-mediated tumor control. However, under conditions of persistent antigen exposure, such as chronic viral infection and cancer, antigen-experienced CD8^+^ T cells progressively lose proliferative capacity, effector function, and long-term persistence, ultimately acquiring a dysfunctional state known as T-cell exhaustion. First systematically defined in chronic lymphocytic choriomeningitis virus (LCMV) infection, T-cell exhaustion is now recognized as a distinct differentiation program that emerges in response to sustained antigen receptor signaling and is further reinforced by inhibitory receptor engagement, metabolic stress, and suppressive signals within the tissue microenvironment (1). Exhausted T cells are typically characterized by progressive but non-uniform functional decline, persistent expression of multiple inhibitory receptors, altered transcription factor networks, and stable epigenetic remodeling. Importantly, exhaustion should not be equated with transient activation, senescence, or simple hyporesponsiveness. Instead, it represents a specialized adaptive state that enables T cells to persist under chronic stimulation, but at the cost of reduced effector competence. In tumors, this program is further intensified by the hostile tumor microenvironment, where persistent antigen presentation, hypoxia, nutrient competition, lactate accumulation, and suppressive cytokines collectively reinforce dysfunction and limit durable immune control. Recent work has also demonstrated that exhausted T cells are not a homogeneous population, but comprise multiple developmental states with distinct molecular features, anatomical localization, and functional potential (2). For example, progenitor exhausted T cells (Tpex) are preferentially localized in or around tertiary lymphoid structures (TLSs) and tumor stroma, where they maintain self-renewal capacity, while terminally exhausted T cells (Texterm) predominantly infiltrate the hypoxic tumor parenchyma (3). The clinical relevance of T-cell exhaustion has become increasingly evident in modern oncology. Immune checkpoint blockade (ICB), especially therapies targeting the PD-1/PD-L1 axis, can partially reinvigorate tumor-reactive T cells and induce meaningful clinical benefit in a subset of patients. Nevertheless, many patients exhibit primary resistance or eventually develop acquired resistance, indicating that checkpoint blockade alone is often insufficient to restore durable antitumor immunity. One major reason is that exhausted T-cell subsets differ substantially in their proliferative potential, developmental plasticity, and susceptibility to therapeutic reinvigoration. In particular, progenitor-like exhausted T cells have emerged as a key response-associated population, whereas terminally exhausted cells are more strongly constrained by stable transcriptional and epigenetic programs and are less amenable to functional recovery (4). Although the biology of T-cell exhaustion has been widely reviewed, existing discussions often emphasize either molecular mechanisms or therapeutic modalities without sufficiently integrating exhaustion-state hierarchy with therapeutic responsiveness (5). Furthermore, many frameworks treat exhaustion as a uniform barrier rather than a spectrum of actionable and non-actionable states. The central clinical problem is no longer simply how to “reverse” exhaustion in general, but how to identify which exhausted T-cell states remain therapeutically actionable and how different interventions should be matched to those states. In this review, we aim to provide a comprehensive, state-oriented framework for understanding T-cell exhaustion in tumor immunology. By differentiating this state-oriented model from traditional binary views of functional versus dysfunctional T cells, we systematically review the developmental continuum of exhausted T-cell subsets, core molecular mechanisms, and their specific implications for major immunotherapies including ICB, adoptive cell therapy (ACT), and emerging combination strategies. We emphasize that future progress relies on precision approaches that preserve progenitor pools, prevent terminal differentiation, and remodel the suppressive TME.

## 2 Biological characteristics of T-cell exhaustion

### 2.1 Functional characteristics

Under chronic antigen exposure in infection and cancer, CD8^+^ T cells progressively lose proliferative capacity, cytokine production, and durable effector function and enter heterogeneous exhausted states. This functional decline is gradual rather than uniform across all settings. Reduced IL-2 production and impaired proliferative potential are generally early features, whereas more advanced dysfunction is associated with diminished polyfunctionality, including reduced TNF-α and often IFN-γ production, together with impaired long-term target-cell control. Importantly, exhaustion does not imply complete functional silence, because some dysfunctional or intermediate exhausted subsets can still retain residual cytotoxic programs under specific conditions (6).

### 2.2 Phenotypic characteristics

The defining phenotypic feature of exhausted T cells is sustained co-expression of multiple inhibitory receptors together with reduced proliferative and effector capacity. Expression of a single checkpoint receptor is insufficient, because recently activated T cells may also transiently express molecules such as PD-1. In contrast, persistent co-expression of receptors including PD-1, LAG-3, TIM-3, CTLA-4, and TIGIT more reliably reflects an exhausted state, particularly when accompanied by functional impairment. In many contexts, broader inhibitory-receptor co-expression is associated with deeper dysfunction, although this relationship remains influenced by activation status, tissue environment, and developmental stage (7).

## 3 Exhausted T-cell subsets

### 3.1 The exhaustion continuum

Under persistent antigenic stimulation within the TME, activated CD8^+^ T cells progressively diverge from canonical memory differentiation and enter an exhaustion trajectory. Rather than representing a collection of fully discrete and static subpopulations, T-cell exhaustion is increasingly understood as a hierarchically organized and epigenetically constrained developmental continuum (8). Although different nomenclatures remain in use, a biologically informative framework broadly resolves this continuum into three major states: progenitor exhausted T cells (Tpex), intermediate or transitory exhausted T cells (Texint), and terminally exhausted T cells (Texterm). In this context, subset assignment is most robust when anchored in lineage-associated transcriptional programs and epigenetic features, whereas individual surface markers are often context dependent and should be interpreted cautiously. The defining functional, spatial and the therapeutic characteristics of these three major exhausted T cell states are summarized in **Table 1**.

**Table 1:** Representative features of major exhausted CD8+ T-cell states.

Subset	Core transcription factors	Representative surface molecules	Effector characteristics	Spatial localization	Therapeutic relevance	Predominant evidence base	References
Tpex	TCF-1, LEF1, MYB, ID3	PD-1, SLAMF6, CXCR5, CD62L (context dependent)	Limited immediate cytotoxicity; self-renewal; proliferative reserve	Stromal or lymphoid-like niches; TLS-associated regions	Principal reservoir sustaining PD-1 responsiveness	Chronic infection models; mouse tumor models; human tumor analyses	(2, 3, 9–12)
Texint/transitory exhausted cells	Reduced TCF-1; T-bet, Zeb2, Id2, Prdm1	CX3CR1, KLRG1, variable PD-1, GZMB, PRF1	Transitional cytotoxic amplification; partial effector-like activity	Intermediate distribution between stromal and parenchymal regions	May contribute to short-lived cytotoxic output after reinvigoration	Mainly preclinical studies, with support from human profiling studies	(9, 11, 13–15)
Texterm	TOX, NR4A family, NFAT, EOMES	High PD-1, TIM-3, CD39, multiple co-inhibitory receptors	Limited proliferation; fixed dysfunctional program; poor reversibility	Tumor parenchyma and non-lymphoid tumor regions	Weak responsiveness to checkpoint blockade alone	Chronic infection models; mouse tumor models; human tumor analyses	(2, 3, 16–19)

#### 3.1.1 Progenitor exhausted T cells (Tpex)

Tpex cells constitute the stem-like compartment of the exhaustion lineage and are most consistently defined by expression of TCF-1, encoded by Tcf7, together with self-renewal capacity and the ability to generate more differentiated exhausted progeny. Although markers such as CD62L, CXCR5, or SLAMF6(Ly108) may help enrich this population in specific settings, none should be regarded as a universal identifier across chronic infection, hematologic malignancies, and solid tumors (9). Functionally, Tpex cells typically display limited immediate cytotoxicity but retain substantial proliferative and developmental potential. Importantly, experimental and clinical studies indicate that Tpex cells are the principal population sustaining responsiveness to PD-1 pathway blockade, and the abundance of this compartment is closely linked to the durability of checkpoint-based antitumor responses (9–11). For example, in melanoma patients, a higher frequency of TCF-1^+^ PD-1^+^ T-cells is predictive of positive clinical outcomes following anti-PD-1 therapy (12).

#### 3.1.2 Intermediate/transitory exhausted T cells (Tex^int^)

As progenitor exhausted cells lose TCF-1 and undergo further differentiation, they acquire an intermediate exhausted state characterized by increased cytotoxic programming and features often associated with effector-like exhausted cells (Texeff), including expression of CX3CR1, KLRG1, GZMB, and PRF1, together with transcriptional regulators such as T-bet, Zeb2, Id2, and Prdm1 (11, 13, 14). This population corresponds to a Ly108^-^CD69^-^ subset. However, current evidence suggests that many of these cells are better interpreted as a differentiating or transitional state within the exhaustion continuum rather than as a fully independent, stable lineage. This distinction has therapeutic implications. Checkpoint blockade appears to act predominantly on the progenitor-like compartment, promoting proliferative expansion and differentiation into more cytotoxic descendants (9). These dynamics likely contribute to the transient increase in antitumor effector activity observed after PD-1 blockade, while continued antigen exposure and the persistent suppressive TME may still constrain long-term durability and favor progression toward more terminally fixed dysfunctional states (6, 15).

#### 3.1.3 Terminally exhausted T cells

**Figure 1:**
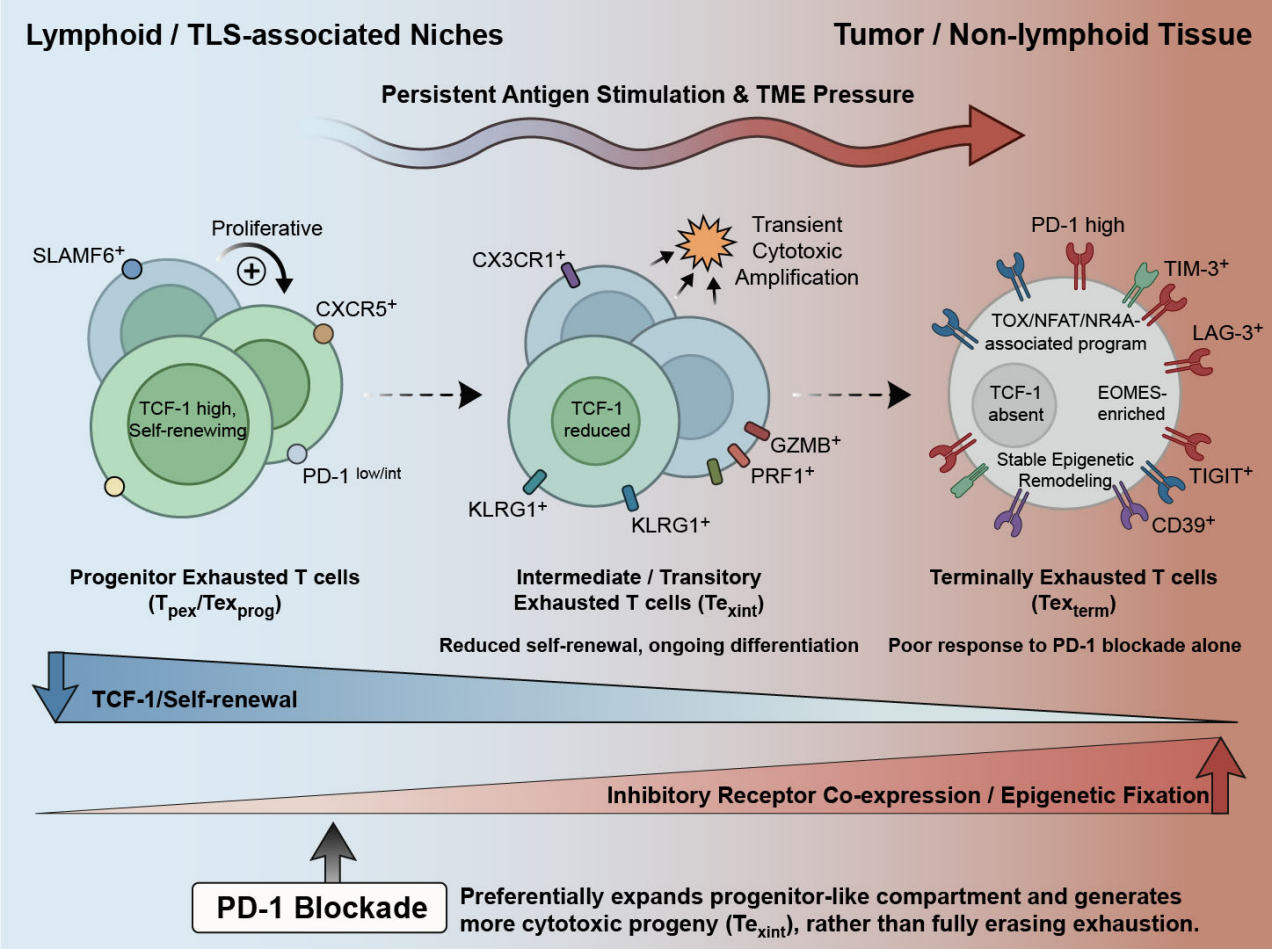
T-cell exhaustion across major immunotherapeutic modalities. Exhausted CD8^+^ T cells are organized along a developmental continuum comprising progenitor exhausted (Tpex/Tex^prog^), intermediate/transitory exhausted (Tex^int^), and terminally exhausted (Tex^term^) states. Across this trajectory, TCF-1 expression, self-renewal capacity, and proliferative potential progressively decline, whereas inhibitory receptor co-expression, TOX-associated programming, and functional constraint increase. Progenitor-like exhausted cells are enriched in lymphoid or lymphoid-like niches, whereas more differentiated exhausted states preferentially accumulate in tumor and non-lymphoid tissues. PD-1 blockade acts predominantly on the progenitor-like compartment, promoting its expansion and the generation of more cytotoxic progeny rather than fully erasing the exhaustion program.

Texterm cells represent the most differentiated end of the exhaustion spectrum under chronic antigen stimulation. These cells have lost TCF-1 expression and are dominated by an exhaustion-associated transcriptional program involving TOX, EOMES, NFAT, and NR4A family members. These TCF-1^-^ cells commonly co-express multiple inhibitory receptors, including PD-1, TIM-3, and CD39, and exhibit limited proliferative capacity despite retaining, in some contexts, residual cytotoxic features. More fundamentally, Texterm cells are distinguished by deeply entrenched transcriptional and epigenetic remodeling rather than by surface phenotype alone. Persistent TOX- and NFAT-associated programs, together with exhaustion-linked chromatin remodeling and *de novo* DNA methylation, help stabilize this dysfunctional state and limit its responsiveness to checkpoint blockade alone (17, 18). Importantly, recent studies highlight that Texterm are not entirely functionally inert; they possess residual cytotoxic potential and their presence is strongly associated with tumor immunogenicity markers such as microsatellite instability (MSI), high tumor mutational burden (TMB), and neoantigen load (20).Thus, terminal exhaustion is best viewed not simply as functional hyporesponsiveness, but as a highly constrained cell state that remains difficult to fully reprogram therapeutically. As shown in **Figure 1**, the exhaustion continuum is closely linked to therapeutic responsiveness across major immunotherapeutic modalities.

### 3.2 Heterogeneity of exhausted T-cell subpopulations

**Figure 2:**
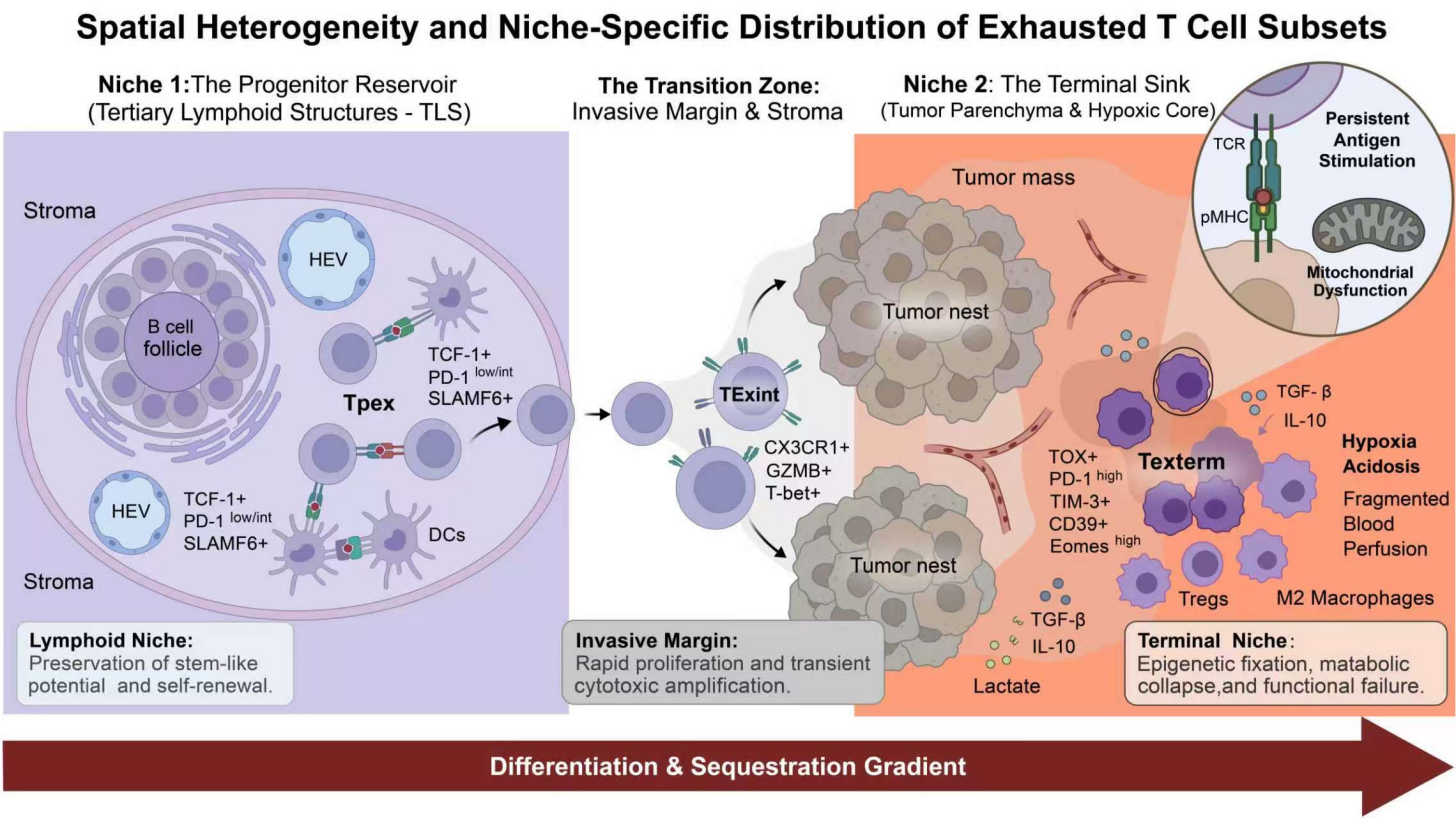
Spatial heterogeneity and niche-dependent organization of exhausted T-cells within the tumor microenvironment. Progenitor exhausted Tcells (Tpex) are preferentially enriched in stromal regions and in or around tertiary lymphoid structures (TLSs), functioning as a reservoir for less differentiated states. Mature TLSs further support intratumoral immune activation. In contrast, terminally exhausted T cells (Texterm) predominantly infiltrate the hypoxic tumor parenchyma, frequently forming tightly packed clusters adjacent to metabolically stressed antigen-presenting cells (APCs). The integration of multiplex imaging allows for the clinical assessment of these spatial metrics to predict therapeutic responsiveness.

T-cell exhaustion is a highly heterogeneous and dynamic process. Exhausted T-cell heterogeneity is shaped by a complex regulatory network integrating chronic antigen stimulation, transcriptional and epigenetic remodeling, metabolic constraints, and microenvironmental signals (21). Consequently, exhausted T cells comprise multiple developmental states with distinct molecular features, anatomical distributions, and functional capacities. In general, this heterogeneity can be understood along a continuum extending from Tpex, through intermediate or transitory states, to Texterm. Recent high-dimensional profiling reveals that even within the terminal exhaustion phase, multiple subsets exist (e.g., TOXhigh, ZNF683high, and CD38high populations), highlighting that exhaustion encompasses multiple terminal states rather than a single uniform phenotype (20). Spatial heterogeneity further contributes to this complexity (**Figure 2**). Precursor-like exhausted CD8^+^ T cells, particularly TCF1^+^ subsets, are preferentially enriched in stromal regions and in or around tertiary lymphoid structures (TLSs), whereas more differentiated exhausted T cells are more abundant within the tumor parenchyma (3, 19). Recent evidence has shown that CD39^+^PD-1^+^CD8^+^ precursor exhausted T cells are concentrated within and around TLSs, supporting the view that lymphoid-like niches serve as reservoirs for less differentiated exhausted states. In addition, mature TLSs have been linked to intratumoral immune activation, whereas the functional properties of immature or poorly organized TLSs appear to be more variable and may be influenced by local immunosuppressive stromal elements (3). Conversely, Ttex cells often form tightly packed clusters adjacent to hypoxic pockets and metabolically stressed antigen-presenting cells (APCs) within the tumor core, creating a stromal-metabolic ecosystem that reinforces exhaustion fixation (20).

Exhaustion programs also vary across tumor types. In aggressive breast cancer, including triple-negative and other ERα-negative contexts, tumor-derived extracellular vesicles have been reported to transfer TGF-β type II receptor (TβRII) to CD8^+^ T cells, thereby activating SMAD3, which cooperates with TCF1 to promote an exhaustion-associated program (22). In pediatric B-cell acute lymphoblastic leukemia (B-ALL), dysfunctional CD4^+^ bone marrow T cells, particularly TIM-3^+^ subsets, have been associated with relapse risk, suggesting that clinically relevant T-cell dysfunction in this disease may be especially prominent in the CD4^+^ compartment (23). By contrast, in multiple myeloma, classical antigen-driven terminal exhaustion may be less dominant than in many solid tumors, in part because impaired antigen presentation and broader immune dysfunction also shape T-cell failure and may contribute to the limited efficacy of ICB monotherapy (24, 25).

## 4 Mechanisms underlying T-cell exhaustion

**Figure 3:**
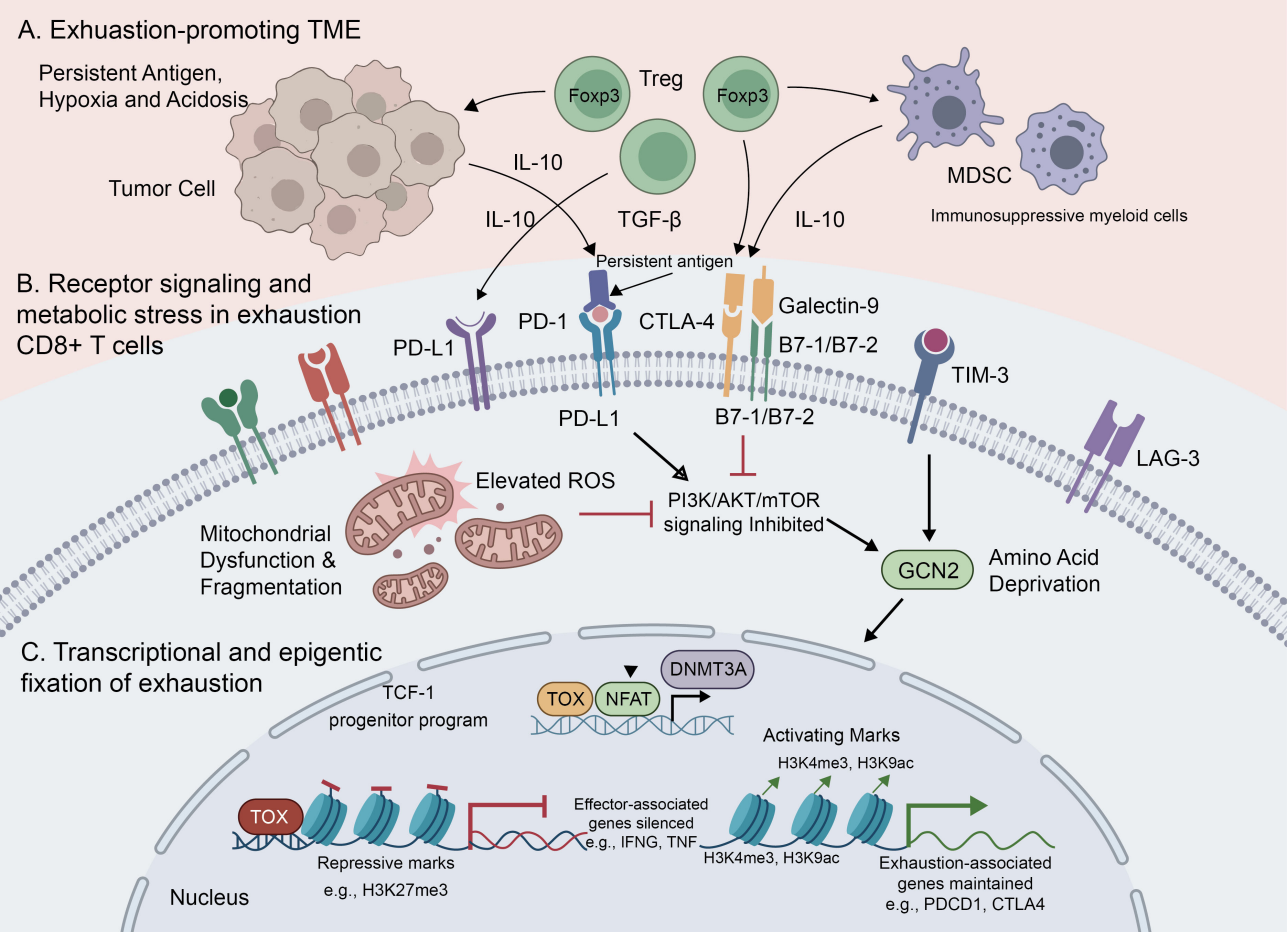
Mechanisms underlying T-cell exhaustion in the tumor microenvironment. Persistent antigen stimulation within the tumor microenvironment (TME) drives chronic TCR signaling in CD8^+^ T cells and initiates a progressive exhaustion program. **(A)** The exhaustion-promoting TME is characterized by persistent antigen exposure, hypoxia, acidosis, suppressive cytokines such as IL-10 and TGF-β, and immunosuppressive cell populations including regulatory T cells (Tregs) and myeloid-derived suppressor cells (MDSCs). **(B)** At the membrane and cytoplasmic levels, sustained co-inhibitory receptor signaling, including PD-1, CTLA-4, TIM-3, and LAG-3, together with amino acid deprivation, PI3K-AKT-mTOR inhibition, mitochondrial dysfunction, and reactive oxygen species (ROS) accumulation, progressively impairs T-cell activation, proliferation, and effector function. **(C)** In the nucleus, chronic stimulation activates NFAT-TOX-centered transcriptional programs and exhaustion-associated epigenetic remodeling, including DNMT3A-mediated regulation, repressive histone modifications at effector-associated loci, and maintenance of exhaustion-related gene expression. TCF-1-associated progenitor programs preserve a precursor-like exhausted state, whereas progressive transcriptional and epigenetic fixation promotes terminal dysfunction and limits reversibility.

From a mechanistic perspective, T-cell exhaustion is not caused by a single suppressive signal, but instead results from the progressive integration of persistent antigenic stimulation, transcriptional and epigenetic rewiring, receptor-mediated inhibitory reinforcement, metabolic insufficiency, and tumor-microenvironmental suppression. Chronic TCR signaling serves as the initiating axis of exhaustion, whereas transcription factor networks determine lineage commitment, epigenetic remodeling stabilizes the dysfunctional state, and inhibitory receptor signaling, metabolic stress, and the TME collectively sustain and amplify dysfunction over time (26). As summarized in **Figure 3**, these mechanisms operate in a coordinated manner to initiate, stabilize, and sustain the exhausted state.

### 4.1 Intracellular transcriptional and epigenetic regulation

#### 4.1.1 Transcription factor networks

During the early stage of exhaustion, progenitor exhausted T cells (Tpex) are maintained by a stemness-associated transcriptional network centered on TCF-1, encoded by the Tcf7 gene. TCF-1 preserves self-renewal capacity, restrains premature terminal differentiation, and supports the long-term maintenance of a responsive precursor pool under persistent antigen exposure. Importantly, TCF-1 does not function in isolation, but cooperates with transcription factors such as LEF1, MYB, and ID3 to stabilize progenitor-like identity and preserve developmental plasticity (2, 4, 27). As chronic stimulation persists, however, T cells progressively engage an exhaustion-inducing transcriptional axis driven by sustained TCR signaling. Persistent calcium influx activates the calmodulin–calcineurin–NFAT pathway, and, under conditions in which canonical AP-1 cooperation is limited, NFAT preferentially induces exhaustion-associated regulators such as TOX and members of the NR4A family, including NR4A1, NR4A2, and NR4A3 (2, 16, 28, 29). TOX and NR4A factors cooperate to impose and reinforce the exhausted transcriptional program, promoting sustained expression of inhibitory receptors while constraining effector-associated genes and limiting functional recovery. Importantly, TOX should not be viewed merely as a suppressor of effector function, but rather as a lineage-stabilizing factor that enables adaptation to chronic stimulation while simultaneously restricting effector plasticity (1, 16, 30, 31).

Additional transcription factors, including BATF, IRF4, and Blimp-1, further strengthen this differentiated dysfunctional state. Under chronic antigenic stimulation, these factors contribute to repression of memory-associated programs and promote progression toward terminal exhaustion of CD8^+^ T cells (32, 33). Likewise, changes in the relative balance of T-bet- and Eomes-associated programs accompany the transition from less differentiated toward more terminally exhausted states. In general, T-bet is more closely linked to preservation of effector competence and restraint of excessive inhibitory receptor expression, whereas Eomes is more often associated with late-stage differentiation and maintenance of a residual but restricted cytotoxic program (34, 35).

#### 4.1.2 Epigenetic reprogramming

Although transcription factor networks initiate and shape the exhaustion program, epigenetic remodeling provides a principal basis for its long-term stabilization and limited reversibility. Exhausted T cells acquire a distinct epigenetic landscape characterized by so-called “epigenetic scars,” including altered chromatin accessibility, histone modifications, DNA methylation, and broader chromatin reorganization (4, 28).

Within progenitor exhausted T cells, loci associated with stemness and persistence remain relatively accessible and enriched in activating histone marks such as H3K4me3 and H3K27ac. As cells progress toward terminal exhaustion, however, chromatin regions associated with effector genes become progressively restricted, whereas exhaustion-related loci remain accessible and transcriptionally active. TOX-centered regulatory complexes contribute to this process by coordinating chromatin remodeling and helping maintain accessibility at exhaustion-associated genes while restricting reactivation of canonical effector programs (26, 30). In parallel, persistent stimulation promotes DNMT3A-mediated *de novo* DNA methylation, which further consolidates the dysfunctional state and limits the ability of exhausted T cells to revert to classical effector or memory fates (1, 16, 36). Chromatin-remodeling complexes such as PBAF may also regulate progenitor maintenance and differentiation transitions, although these regulators are better viewed as modulators of exhaustion-state architecture than as sole determinants of lineage commitment (26, 37). Collectively, these findings help explain why therapeutic reinvigoration, including checkpoint blockade, is often partial rather than fully restorative.

### 4.2 Sustained upregulation of inhibitory receptors

Sustained inhibitory receptor signaling is a major reinforcing mechanism of T-cell exhaustion. Under physiological conditions, receptors such as PD-1, CTLA-4, LAG-3, TIM-3, and TIGIT are essential for maintaining immune tolerance and limiting excessive inflammation. However, under chronic pathological stimulation, these pathways remain persistently engaged and progressively suppress T-cell activation, proliferation, cytokine production, and survival (2). Importantly, inhibitory receptors should not be interpreted merely as phenotypic markers, nor as the sole origin of exhaustion, but as receptor-mediated circuits that reinforce dysfunction once chronic antigen-driven lineage programming has already been established.

Mechanistically, PD-1 primarily recruits SHP-2 following phosphorylation of its cytoplasmic motifs, thereby suppressing proximal TCR/CD28 signaling and downstream PI3K/AKT and ERK pathways. In some contexts, SHP-1 may also contribute to PD-1-mediated inhibitory signaling (38, 39). CTLA-4 limits co-stimulation by competing with CD28 for CD80 and CD86, whereas TIM-3 and TIGIT provide additional nonredundant inhibitory inputs through their respective ligands, including galectin-9 and CD155. Because exhausted T-cells frequently co-express multiple inhibitory receptors, blockade of one pathway often triggers compensatory reliance on others, underscoring the redundancy and adaptability of exhaustion-associated signaling circuits (16, 40, 41).

### 4.3 Metabolic insufficiency and mitochondrial dysfunction

Metabolic dysfunction is another central feature of exhausted T cells, particularly within tumors, where malignant cells and suppressive stromal or myeloid populations create profound nutrient competition. One major consequence is reduced access to glucose, which compromises glycolytic flux and anabolic fitness in tumor-reactive CD8^+^ T cells. This defect is further reinforced by PD-1 signaling, which suppresses the PI3K/AKT/mTOR pathway and thereby limits glucose uptake and metabolic support for proliferation and effector responses. As a result, exhausted T cells lose the bioenergetic flexibility required to sustain durable antitumor activity (42).

Mitochondrial insufficiency further aggravates this state. Exhausted T cells commonly exhibit impaired mitochondrial fitness, abnormal mitochondrial morphology, and reduced oxidative capacity, all of which constrain long-term persistence and function (16, 43). Recent studies highlight that Ttex undergo mitochondrial dysfunction driven by PGC1α suppression, impaired oxidative phosphorylation, and reactive oxygen species (ROS) accumulation (20). Damaged mitochondria also generate excessive reactive oxygen species (ROS), promoting stress responses, apoptosis, and further functional decline. In this setting, shifts toward alternative metabolic pathways, including greater dependence on lipid metabolism and fatty acid oxidation, may provide incomplete compensation but often fail to restore full fitness under chronic stress (44, 45). In some contexts, lipid peroxidation and ferroptosis-related stress may further aggravate dysfunction, although their exact contribution appears to be context dependent.

Amino acid deprivation represents a third major metabolic axis. Tumor cells and myeloid-derived suppressor cells (MDSCs) consume arginine and tryptophan through high expression of Arg1 and indoleamine 2,3-dioxygenase (IDO), while simultaneously generating immunosuppressive metabolites that activate stress-response pathways in T cells, including GCN2-associated signaling and inhibition of mTORC1. These changes impair cell-cycle progression, biosynthetic competence, and effector function (45, 46).

### 4.4 Regulation by the tumor microenvironment

The tumor microenvironment sustains T-cell exhaustion not through a single suppressive factor, but as an integrated ecosystem that provides persistent antigenic stimulation, defective priming conditions, suppressive cytokines, metabolic restriction, and inhibitory cellular interactions. Persistent antigen presentation is one of the major upstream drivers of exhaustion in both chronic infection and cancer. In tumors, dendritic-cell composition and function are often altered, with reduced co-stimulation, increased inhibitory ligand expression, and impaired support for productive T-cell priming. Under these conditions, antigen presentation becomes skewed toward maintaining chronic stimulation without effective clearance, thereby favoring exhaustion-prone differentiation (21).

Soluble inhibitory mediators provide an additional layer of suppression. Immunoregulatory cytokines such as IL-10 and TGF-β accumulate progressively within the TME and dampen antitumor immunity by impairing APC function, reducing inflammatory cytokine production, and directly constraining T-cell proliferation and effector differentiation (47, 48). These cytokines do not independently create exhaustion, but they strongly reinforce and stabilize the dysfunctional state once chronic stimulation is established (48, 49).

Biophysical and metabolic stresses within tumors also contribute substantially. The abnormal vasculature of solid tumors creates a profoundly hypoxic milieu, which impairs oxidative phosphorylation, reshapes metabolic and transcriptional programs, and exacerbates mitochondrial stress and ROS accumulation (50, 51). In parallel, persistent aerobic glycolysis by tumor cells leads to extracellular lactate accumulation and microenvironmental acidification. Elevated extracellular lactate can hinder lactate efflux from activated T-cells, disrupt intracellular pH homeostasis, and suppress proliferation and cytokine production. These changes may also contribute to broader epigenetic remodeling associated with dysfunctional immune states, although the precise role of mechanisms such as histone lactylation in exhausted T cells remains under active investigation (52, 53).

Finally, multiple immunosuppressive cell populations cooperate to maintain exhaustion within tumors. Regulatory T cells (Tregs) restrict IL-2 availability and suppress co-stimulation, most notably through high-affinity CD25 expression and CTLA-4-dependent modulation of APC function (1). Importantly, MDSCs have emerged as key extrinsic drivers of immune suppression and therapy resistance. MDSCs generate inhibitory metabolites and reactive species, express PD-L1, and exploit multiple amino acid metabolic pathways to establish and sustain immunosuppression, thereby directly contributing to T cell exhaustion and reinforcing the notion that therapy resistance is not solely T-cell intrinsic but also myeloid-driven (54, 55). Tumor-associated macrophages (TAMs), particularly those polarized toward an M2-like phenotype, further amplify exhaustion through suppressive cytokines, chemokines, and inhibitory ligand expression (56). Together, these cellular components reduce the likelihood that tumor-reactive T cells can recover durable effector competence *in situ*.

## 5 Therapeutic consequences of the exhaustion continuum in cancer immunotherapy

The major ways in which T-cell exhaustion constrains current cancer immunotherapy are summarized in **Table 2**.

**Table 2:** Major immunotherapeutic modalities influenced by T-cell exhaustion: barriers, intervention strategies, and translational limitations.

Therapeutic strategy	How exhaustion limits efficacy	Representative strategies	Therapeutic implications and major limitations (References)
PD-1/PD-L1 or CTLA-4 blockade	ICB can partially reinvigorate exhausted T cells, but cannot fully erase exhaustion-associated epigenetic programs; benefit is reduced when terminal exhaustion predominates.	PD-1/PD-L1 blockade; CTLA-4 blockade; dual checkpoint blockade.	Preferentially benefits progenitor-like exhausted T cells and transiently enhances effector output, but durability is limited and toxicity increases with combination blockade (4, 12, 57).
Adoptive cell therapy (ACT, including CAR-T, TCR-T, and TIL therapy)	Infused T cells may already be differentiated or become progressively dysfunctional after transfer; in engineered products, tonic signaling, chronic antigen exposure, and suppressive TME conditions further accelerate exhaustion and reduce persistence.	Selection or enrichment of less differentiated T cells; ex vivo conditioning; CAR redesign; 4-1BB costimulation; CRISPR-based editing (e.g., CD5 deletion); stem-like TIL enrichment.	Durable benefit depends on preserving stem-like or persistent T-cell states, but antigen escape, product heterogeneity, poor trafficking in solid tumors, manufacturing complexity, and on-target/off-tumor toxicity remain major barriers (58–62).
Therapeutic cancer vaccines	Vaccine-induced T cells may rapidly become dysfunctional in the suppressive TME, limiting expansion and sustained killing.	Neoantigen vaccines; mRNA vaccines; vaccine plus ICB.	Can broaden tumor-specific immunity and replenish responsive T-cell pools, but monotherapy activity is often limited in established tumors (63–65).
Cytokine-based interventions	Suppressive cytokine milieus reinforce dysfunction, while inadequate survival signals impair maintenance of tumor-reactive T cells.	IL-15-based therapy; cytokine engineering; blockade of suppressive cytokine pathways.	May support survival and progenitor-like pools, but specificity and systemic toxicity remain concerns; Anktiva shows clinical translation in a defined bladder cancer setting (66–68).
Metabolic reprogramming	Hypoxia, nutrient deprivation, lactate accumulation, and mitochondrial dysfunction reduce T-cell fitness.	PGC-1α-associated support; FAO/PPAR-related approaches; ex vivo metabolic conditioning; MDSC targeting.	May improve mitochondrial fitness and resistance to dysfunction, but effects are highly context dependent and clinical validation remains limited (43, 55, 69, 70).
Combination strategies	No single modality fully addresses the multiple layers of exhaustion biology.	ICB plus vaccine; ICB plus cytokine agonists; ICB plus metabolic support; ICB plus radiotherapy/chemotherapy; oncolytic virotherapy plus ICB.	Potentially provides complementary activity across priming, reinvigoration, and TME remodeling, but toxicity, sequencing, and biomarker selection remain unresolved (4, 71–73).

ACT, adoptive cell therapy; CAR-T, chimeric antigen receptor T-cell therapy; CTLA-4, cytotoxic T-lymphocyte-associated protein 4; ICB, immune checkpoint blockade; PD-1, programmed cell death protein 1; PD-L1, programmed death-ligand 1; TIL, tumor-infiltrating lymphocyte; TME, tumor microenvironment; Tpex, progenitor exhausted T cell; Texterm, terminally exhausted T cell.

### 5.1 Immune checkpoint blockade

Immune checkpoint blockade (ICB), including antibodies targeting PD-1, PD-L1, and CTLA-4, remains one of the most widely used and clinically important forms of cancer immunotherapy. However, the efficacy of ICB is profoundly shaped by the differentiation state and plasticity of tumor-reactive T cells (5, 57). Although PD-1 pathway blockade can partially reinvigorate exhausted T cells and induce tumor regression in a subset of patients, these responses are frequently incomplete and often lack durability. A major reason is that checkpoint inhibition does not fully erase the transcriptional and epigenetic programs that stabilize the exhausted state. Instead, current evidence indicates that the principal responders to PD-1 blockade are progenitor-like exhausted T cells, particularly the TCF-1^+^ compartment, which retains proliferative potential and can generate more differentiated effector-like progeny after treatment (4). By contrast, when the intratumoral T-cell pool is dominated by terminally exhausted, epigenetically constrained cells, PD-1 blockade alone is often insufficient to restore durable antitumor immunity. Thus, the limited durability of ICB reflects not only incomplete functional reinvigoration, but also the persistence of exhaustion-associated chromatin states that restrict long-term cellular plasticity (4, 57, 74).

Mechanistically, persistent PD-1 engagement attenuates T-cell activation by suppressing proximal TCR and CD28 signaling and by constraining downstream pathways involved in proliferation, survival, and effector differentiation. Ligation of PD-1 by PD-L1 or PD-L2 promotes phosphorylation of the cytoplasmic tail of PD-1, especially the immunoreceptor tyrosine-based switch motif, leading predominantly to SHP-2 recruitment and assembly of an inhibitory signaling complex that dampens PI3K–AKT and RAS–MEK–ERK signaling. Consequently, chronic PD-1 signaling progressively limits cytokine production, cytotoxicity, and proliferative fitness. In this context, PD-1 blockade should be understood primarily as a strategy that releases functional restraint on responsive exhausted T-cell populations and amplifies a regenerative precursor pool, rather than as an intervention that fully reverses terminal exhaustion (38, 57, 75).

CTLA-4 blockade has related but non-identical biology. Unlike PD-1 inhibition, which acts mainly on chronically stimulated T cells within peripheral tissues and tumors, CTLA-4 blockade has stronger effects on priming and co-stimulation in lymphoid tissues and can also alter regulatory T-cell activity within tumors (76). Accordingly, CTLA-4-targeted therapy is best viewed as complementary rather than mechanistically interchangeable with PD-1 blockade. This distinction provides part of the rationale for combined PD-1 and CTLA-4 inhibition, which has shown clinical benefit in several settings but also carries substantially greater immune-related toxicity (1, 5, 57, 74). Because exhausted T cells commonly co-express multiple inhibitory receptors, including LAG-3, TIM-3, and TIGIT, single-checkpoint blockade frequently yields only partial benefit (16, 63, 77). Future progress will therefore likely depend on more precise combination strategies guided by tumor type, T-cell subset composition, and the properties of the local immune microenvironment (16). Clinically, agents targeting PD-1 and CTLA-4 have become foundational components of modern oncology, particularly in melanoma and several other solid tumors (5, 57).

### 5.2 Adoptive cell therapy

Adoptive cell transfer (ACT) exerts antitumor effects through the infusion of autologous or allogeneic immune cells that have been expanded or genetically modified ex vivo. Across ACT platforms, the therapeutic efficacy of the infused product is strongly influenced by the differentiation state of the transferred T cells. Although highly differentiated effector cells may provide immediate cytotoxic activity, durable clinical benefit is more consistently associated with less differentiated, stem-like clonotypes that retain self-renewal capacity, proliferative fitness, and long-term persistence *in vivo* (58). Accordingly, if the infused cell product is dominated by terminally exhausted cells, transient tumor killing may still occur, but sustained expansion and durable disease control are unlikely. Effective therapy therefore requires transferred T cells to preserve persistence, serial proliferative capacity, and the ability to functionally re-expand after antigen encounter (36, 58, 78, 79).

In engineered T-cell therapies, including CAR-T and TCR-T, exhaustion is a major barrier to durable efficacy, especially in solid tumors. In CAR-T cells, dysfunction can begin even before infusion because certain receptor designs promote antigen-independent tonic signaling, often through spontaneous receptor clustering linked to properties of the scFv framework, hinge, or transmembrane domain (58, 78, 79). This chronic basal signaling can drive premature activation and initiate exhaustion programs during ex vivo manufacturing. After infusion, CAR-T cells entering the tumor microenvironment are further exposed to persistent antigen stimulation, hypoxia, nutrient restriction, and immunosuppressive factors such as TGF-β, all of which accelerate functional decline (59). Under these conditions, CAR-T cells progressively upregulate inhibitory receptors, lose cytokine production, display diminished proliferative capacity, and fail to maintain long-term tumor surveillance. Similar limitations also apply to TCR-T cells, particularly in solid tumors with sustained antigen burden, where chronic stimulation drives progressive dysfunction and restricts *in vivo* persistence (59, 80, 81).

**Figure 4:**
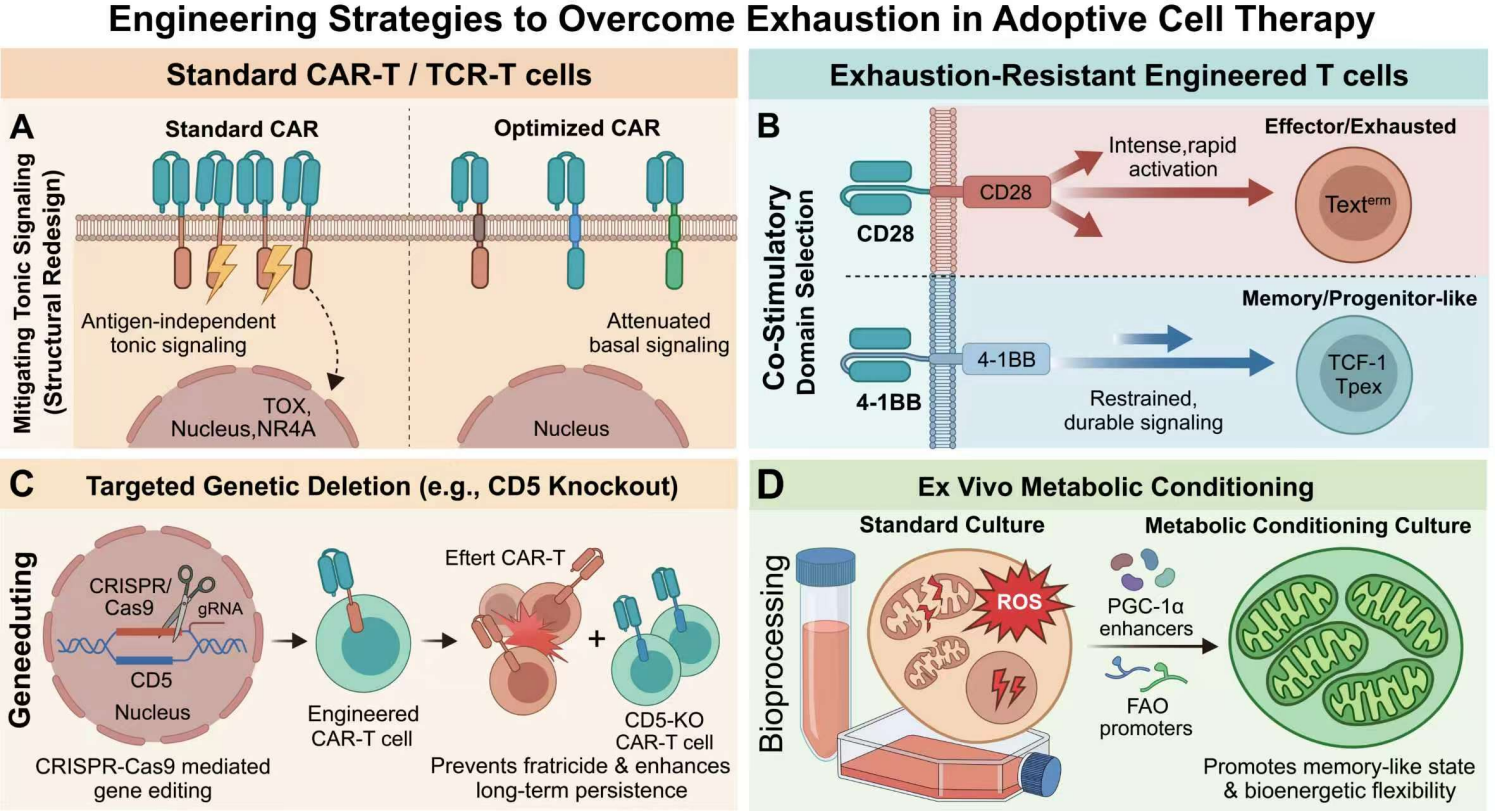
Engineering strategies to mitigate T-cell exhaustion in adoptive cell therapy. Exhaustion in CAR-T cells can be rapidly driven by antigen-independent tonic signaling and suppressive factors within the tumor microenvironment (TME). To enhance CAR-T cell persistence and durability, several engineering approaches are utilized: (1) Structural redesign of the CAR construct (including scFv, hinge, and transmembrane domains) to attenuate constitutive signaling and prevent self-aggregation; (2) Selection of optimal co-stimulatory domains, such as 4-1BB, which provides a more restrained activation profile compared to CD28; and (3) Target-antigen editing via CRISPR, such as CD5 deletion, to prevent fratricide and maintain cellular fitness under chronic stimulation.

These insights have made exhaustion not only a barrier in ACT but also a direct engineering target (**Figure 4**). One major strategy is optimization of receptor architecture to reduce tonic signaling and improve cellular fitness. Structural redesign of CAR constructs, including adjustment of scFv features and hinge-transmembrane configurations, can reduce self-aggregation propensity and attenuate constitutive signaling (82). In addition, the choice of co-stimulatory domain influences exhaustion susceptibility. 4-1BB-based CARs generally provide a more restrained and durable activation profile than CD28-based CARs and are often associated with improved persistence under chronic stimulation. Direct genetic engineering offers another approach, including target-antigen editing strategies such as CD5 deletion to prevent fratricide and enhance CAR-T persistence, although prolonged immunosuppression and on-target/off-tumor toxicity remain important translational limitations (61, 62, 79, 83, 84). A recent phase 1 study evaluating autologous CD5-targeting CAR-T cells with CD5 gene deletion in patients with relapsed/refractory CD5+ T-cell lymphoma provided clinical evidence that CD5 ablation enhances CAR-T persistence and antitumor potency by mitigating exhaustion, highlighting both the promise and the challenges of exhaustion-oriented CAR-T engineering (61, 62).

Tumor-infiltrating lymphocyte (TIL) therapy is similarly shaped by exhaustion, because TILs are isolated directly from tumor tissue and therefore have already undergone prolonged antigen exposure and immunosuppressive conditioning within the tumor microenvironment. Persistent TCR signaling, hypoxia, glucose restriction, and lactate-rich conditions collectively impair mitochondrial fitness and establish exhaustion-associated transcriptional and epigenetic programs before these cells are harvested (85). Importantly, TIL products are highly heterogeneous, and their therapeutic value depends heavily on the subset composition of the infused population (60). Studies in melanoma have identified CD39^-^CD69^-^ TIL populations enriched for a more stem-like state and associated with improved persistence and response after transfer, although such phenotypic definitions should not be generalized uncritically across all tumor types. Overall, enrichment of less differentiated, stem-like T-cell populations appears to be a key determinant of durable ACT-based immunotherapy (58, 60).

### 5.3 Cancer vaccines

T-cell exhaustion is also a major barrier to the clinical efficacy of therapeutic cancer vaccines. Although vaccines are designed to induce or amplify tumor-specific T-cell responses, the T cells generated by vaccination often encounter persistent antigen stimulation and sustained immunosuppressive pressure within the tumor microenvironment, conditions that favor progressive dysfunction. As a result, exhaustion can compromise multiple steps required for vaccine efficacy, including clonal expansion, persistence, and sustained cytotoxic activity. Even when initial priming is successful, vaccine-elicited T cells may fail to expand sufficiently or maintain durable function after entry into the tumor (4). Mechanistically, chronic TCR–NFAT signaling promotes exhaustion-associated regulators such as TOX and members of the NR4A family, reinforcing dysfunctional differentiation and limiting long-term effector fitness (28, 29). These constraints may partly help explain why therapeutic cancer vaccines often show limited activity as monotherapy in established tumors (86, 87).

At the same time, vaccination remains an attractive partner for exhaustion-targeted therapy. Therapeutic cancer vaccines may complement checkpoint blockade not by directly reversing terminal exhaustion, but by broadening antigen-specific immunity, promoting *de novo* or renewed priming, and replenishing more responsive T-cell pools (64). Neoantigen vaccines are particularly promising because tumor-specific somatic mutations provide greater specificity and lower risk of off-tumor autoimmunity than shared tumor-associated antigens. These vaccines can be delivered through peptide, DNA, mRNA, or viral-vector platforms, and mRNA-based personalized vaccines have attracted particular interest because of their flexibility, scalability, and capacity to induce durable T-cell responses (4, 65). Emerging evidence further suggests that neoantigen vaccination can expand proliferating and stem-like PD-1+TCF1+ tumor-specific T cells and synergize with checkpoint inhibition, thereby making vaccines a rational component of combination regimens aimed at overcoming exhaustion-associated dysfunction (63).

### 5.4 Cytokine and metabolic interventions

Because exhausted T cells are shaped not only by inhibitory receptor signaling but also by cytokine imbalance and metabolic stress, supportive interventions targeting these layers have emerged as important therapeutic strategies. Cytokine-based approaches seek either to neutralize suppressive signals or to deliver stimulatory cytokines in a more selective and therapeutically useful manner (88). In chronic infection and cancer, immunosuppressive cytokines such as IL-10 and TGF-β can reinforce T-cell dysfunction, whereas stimulatory cytokines may help preserve or expand more functional T-cell populations (66). Among these, IL-15 has attracted particular interest because it can support proliferation and survival of tumor-reactive T cells, including progenitor-like exhausted populations, while being less prone than conventional IL-2 to expand regulatory T cells (66). This difference reflects, at least in part, the distinct biology of IL-15 trans-presentation through IL-15Rα. The translational relevance of this axis is further supported by the ongoing clinical development of IL-15-based therapeutic strategies (89).

Metabolic reprogramming represents a related but distinct strategy. Exhausted T cells typically reside in environments marked by nutrient competition, chronic hypoxia, lactate accumulation, oxidative stress, and mitochondrial dysfunction (43). These conditions undermine mitochondrial integrity and bioenergetic flexibility, both of which are central to sustained T-cell function. Accordingly, interventions that improve mitochondrial fitness have become a major area of interest. PGC-1α, a key regulator of mitochondrial biogenesis and oxidative metabolism, has emerged as one of the best-supported mechanistic nodes in this space, and related programs involving fatty acid oxidation and PPAR-associated metabolism may also help sustain T-cell fitness under chronic stress (43, 69, 90). However, the benefit of metabolic interventions remains context dependent and likely varies by tumor type, metabolic niche, and differentiation state of the T-cell population. Metabolic manipulation may also be useful during ex vivo manufacture of CAR-T or TCR-T products, where promoting memory-like rather than terminally differentiated states could improve persistence and resistance to exhaustion after infusion (70).

### 5.5 Rational combination strategies

**Figure 5:**
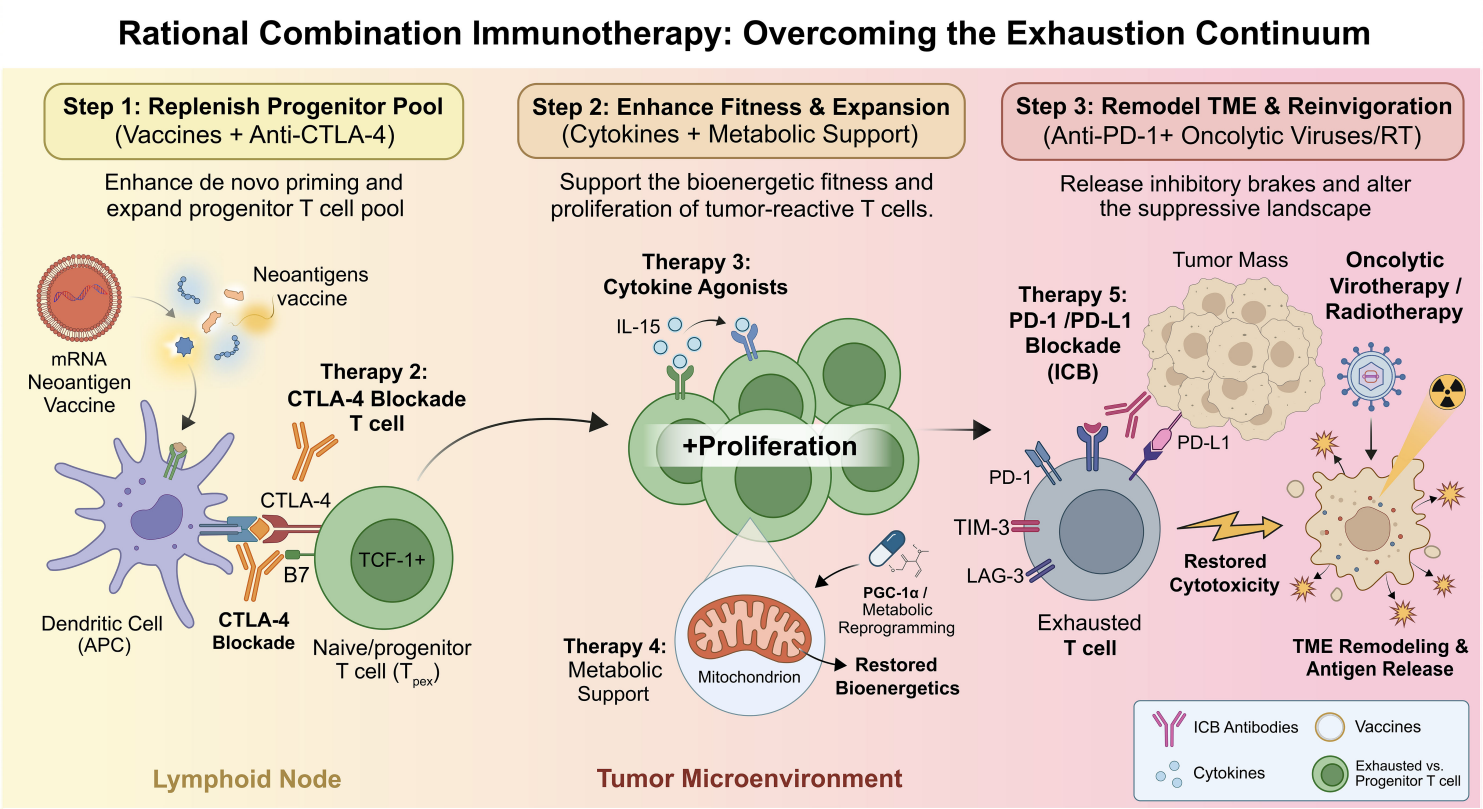
Rational combination strategies targeting the multifactorial T-cell exhaustion continuum. Because T-cell exhaustion is driven by an integrated network of suppressive signals, rational combination therapies must intervene at distinct but complementary levels. Neoantigen vaccines can broaden antigen-specific immunity and replenish the pool of proliferating stem-like tumor-reactive T cells. Immune checkpoint blockade (ICB) acts primarily on the progenitor-like pool to promote expansion and differentiation into cytotoxic progeny. Cytokine interventions (e.g., IL-15) and metabolic reprogramming provide essential survival signals and alleviate bioenergetic barriers. Concurrently, modalities such as oncolytic virotherapy can remodel the suppressive architecture of the tumor microenvironment, further synergizing with T-cell-directed therapies.

No single intervention is likely to fully overcome T-cell exhaustion, because exhaustion is a multifactorial state shaped by inhibitory receptor signaling, transcriptional and epigenetic fixation, altered priming, cytokine imbalance, and metabolic restriction. For this reason, combination therapy has emerged as the most compelling framework for future intervention (4). The logic of combination treatment is not simply intensification, but functional complementarity: one modality may improve priming, another may release inhibitory signaling, another may support T-cell fitness, and yet another may remodel the tumor microenvironment (79). In this view, and as conceptualized in **Figure 5**, effective treatment should aim not at indiscriminate “reversal” of exhaustion, but at selective reinvigoration of responsive populations, preservation or expansion of progenitor-like resource cells, and mitigation of the ecological pressures that drive terminal dysfunction.

Clinically, combined PD-1 and CTLA-4 blockade remains one of the best-established examples of this principle, offering meaningful benefit in selected patients while also increasing immune-related toxicity (71). More experimental combinations are mechanistically attractive because they intervene at distinct but complementary levels of exhaustion biology. Checkpoint blockade plus neoantigen vaccination may replenish the pool of proliferating stem-like tumor-reactive T cells, checkpoint blockade plus cytokine agonists may enhance their expansion and effector differentiation, and checkpoint blockade plus metabolic support may alleviate the mitochondrial and bioenergetic barriers that reinforce persistent dysfunction (66, 91). Radiotherapy and selected chemotherapies may also contribute by inducing immunogenic tumor cell death, increasing antigen availability, and improving responsiveness to immunotherapy in some settings (92). Recent clinical evidence further highlights that oncolytic virotherapy can effectively remodel tumor-immune architecture and establish lasting spatial immune surveillance, providing a novel combination approach to overcome therapy resistance via TME remodeling (73). However, the success of combination therapy will likely depend on more precise matching between therapeutic modality and exhaustion state, including the abundance of TCF-1^+^ precursor-like cells, the depth of epigenetic fixation, and the composition of the tumor immune microenvironment (9, 15, 93).

## 6 Clinical and translational evidence for T-cell exhaustion

Recent translational studies have substantially refined the clinical interpretation of T-cell exhaustion in human cancer. Rather than representing a single dysfunctional endpoint, exhausted CD8^+^ T cells in tumors are now increasingly understood as a hierarchically organized and spatially structured population with distinct degrees of proliferative capacity, lineage plasticity, and therapeutic responsiveness. Accordingly, clinical outcome appears to depend not simply on the abundance of exhausted T cells, but on the balance between progenitor-like resource populations and more terminally constrained states, as well as on the local tissue niches in which these subsets are maintained. This state-dependent view provides an important bridge between mechanistic studies of exhaustion and the variable efficacy of contemporary cancer immunotherapy (9).

### 6.1 Human tumor evidence and spatial organization of exhausted Tcells

High-dimensional analyses of human tumors have shown that exhausted T cells exist as heterogeneous states rather than a single terminal population. Across cancers, these states broadly form a continuum spanning progenitor-like, intermediate, and terminally exhausted subsets, which differ in proliferative capacity and responsiveness to immune checkpoint blockade. Spatial studies further show that progenitor-like exhausted T cells are enriched in lymphoid-like niches (as depicted in **Figure 2**), especially around tertiary lymphoid structures, whereas terminally differentiated exhausted cells are more common in the tumor parenchyma (9). In esophageal cancer, TLS-associated TCF-1^+^ subsets within CD39^+^CD8^+^ exhausted T cells were linked to better response to PD-1 blockade, and similar findings in head and neck cancer connected mature TLS with progenitor-exhausted T-cell programs and stronger intratumoral immune activation. Recent work in renal cell carcinoma also supports the clinical relevance of TLS context and exhausted tissue-resident T-cell states in shaping PD-1 response (3, 94). Together, these findings indicate that exhaustion in human tumors is best understood as a state-dependent and niche-dependent program, with clinical significance determined more by developmental composition, spatial localization, and retained plasticity than by the simple presence of PD-1^+^ dysfunctional T cells (3).

### 6.2 Biomarkers of response and clinical translation

Among proposed biomarkers, progenitor-like exhausted T cells marked by TCF-1-associated programs have emerged as one of the most informative indicators of responsiveness to PD-1-directed therapy. These cells retain proliferative capacity, generate more differentiated progeny after treatment, and appear to constitute the principal compartment mobilized by checkpoint blockade. In contrast, tumors enriched for terminally exhausted populations with stronger inhibitory-receptor co-expression and deeper transcriptional or epigenetic fixation are generally less amenable to durable reinvigoration (9).

Recent studies have highlighted the Ttex/CD8^+^ ratio as a promising biomarker in solid tumors such as colorectal, lung, and esophageal carcinoma, where the prognostic significance of Ttex is highly tumor-context-dependent, shaped by stromal architecture, mutational burden, and progenitor Tpex availability (20). A more useful translational framework therefore integrates differentiation state with spatial organization and local immune ecology rather than relying on single checkpoint markers alone. In particular, the abundance of TCF-1^+^ precursor-like cells, the presence and maturity of tertiary lymphoid structures, and the relative dominance of terminally constrained exhausted states may together provide a more clinically meaningful readout of response potential (3). The advent of multiplex imaging, digital pathology, and AI-driven quantification has facilitated the standardized assessment of these spatial and phenotypic metrics, paving the way for their integration into clinical practice (20). This framework may also help distinguish primary from acquired resistance. Primary resistance may reflect a scarcity of responsive precursor-like cells or a strongly suppressive baseline microenvironment, whereas acquired resistance may emerge after transient reinvigoration as T cells continue to progress toward terminally constrained states (9).

### 6.3 A disease-limiting role of exhaustion in autoimmunity

Human chronic viral infections provided the earliest translational foundation for understanding T-cell exhaustion and established the central role of persistent antigen exposure in driving inhibitory receptor expression, proliferative impairment, and loss of effector fitness. These studies were crucial because they defined exhaustion as a biologically coherent adaptive state rather than nonspecific immune failure, and they continue to inform how exhaustion is interpreted in cancer (95). Consistent with this view, autoreactive CD8^+^ T cells with an exhaustion-like phenotype have been associated with slower disease progression in type 1 diabetes, supporting the idea that exhaustion can function as a disease-limiting program in at least some autoimmune settings (96).

In contrast, observations from autoimmune disease indicate that exhaustion-like programs may, in some settings, restrain pathological tissue damage. McKinney and colleagues reported that a transcriptional signature reflecting CD8 T-cell exhaustion was associated with poor clearance of chronic viral infection but, conversely, with better prognosis in multiple autoimmune diseases (95). This contrast has important translational implications. Although partial reinvigoration of exhausted T cells may be desirable in cancer, indiscriminate elimination of exhaustion-associated restraints could increase the risk of systemic immune toxicity or autoimmune complications. Therefore, the therapeutic objective should not be complete erasure of exhaustion, but selective and context-appropriate redirection of those exhausted T-cell states that remain clinically actionable (95).

## 7 Future research directions and challenges

Despite major advances in cancer immunotherapy, several key barriers still limit efforts to durably overcome T-cell exhaustion. Future progress will depend on more precise definition of exhausted T-cell states, better understanding of which states remain reversibly programmable, and improved ability to predict exhaustion trajectories in individual patients.

### 7.1 Defining exhaustion states more precisely

A major unresolved issue is the lack of a standardized molecular definition of T-cell exhaustion (16). Exhaustion is still frequently inferred from inhibitory receptor expression or generalized dysfunction alone, which can obscure important differences between developmental states (1). Future studies should therefore integrate spatial, epigenetic, metabolic, and functional readouts to establish more consistent and biologically meaningful classification frameworks. However, the value of such approaches will depend on standardized sampling and cross-cohort validation (97).

### 7.2 Overcoming limited reversibility

A second challenge is the limited reversibility of terminal exhaustion. Although immune checkpoint blockade can partially restore T-cell activity, many terminally exhausted cells remain constrained by stable transcriptional and epigenetic programs. Thus, future research should focus on strategies that enhance functional plasticity without causing off-target toxicity or destabilizing T-cell identity (98). Emerging therapeutic strategies targeting Ttex through immune checkpoint combinations, TOX and circRNA-mediated reprogramming, and exhaustion-resistant T cell engineering offer promising avenues to overcome this limitation (20).

### 7.3 Predicting exhaustion trajectories

A third opportunity lies in predictive modeling. As multi-omic datasets continue to expand, AI- and machine learning-based approaches may help identify which patients are more likely to maintain a responsive progenitor-like pool and which are more likely to progress toward terminal exhaustion under standard therapy. The integration of multiplex imaging and digital pathology will further refine these models by incorporating spatial contexts (20). Nevertheless, the clinical utility of these models will require longitudinal data, biological interpretability, and prospective validation (99).

## 8 Conclusion

T-cell exhaustion is increasingly understood not as a fixed and terminal state of dysfunction, but as a dynamic and hierarchically organized differentiation program that shapes the quality and durability of antitumor immunity. This shift in perspective has important implications for cancer immunotherapy, as clinical responses appear to depend less on the overall abundance of exhausted T cells and more on the balance, plasticity, and spatial distribution of distinct exhaustion states within the tumor microenvironment. Current immunotherapies, including immune checkpoint blockade, act primarily by mobilizing progenitor-like exhausted T cells, whereas more terminally differentiated populations remain constrained by stable transcriptional and epigenetic programs, which likely underlies the limited durability of many responses. Viewed in this context, future strategies may benefit from moving away from attempts to broadly reverse exhaustion and instead focus on selectively sustaining and redirecting T-cell differentiation, including preserving progenitor-like pools, limiting progression toward terminal states, and alleviating the metabolic and immunosuppressive pressures within the tumor microenvironment. A more precise understanding of T cell exhaustion in its molecular, cellular, and tissue context will be important for developing more effective immunotherapies and achieving longer-lasting clinical benefit.
